# Celebrating Todd Marder: 65th Birthday and His Contributions to Inorganic Chemistry

**DOI:** 10.3390/molecules26040776

**Published:** 2021-02-03

**Authors:** Ashok Kakkar

**Affiliations:** Department of Chemistry, McGill University, 801 Sherbrooke St. West, Montreal, QC H3A 0B8, Canada; ashok.kakkar@mcgill.ca

Professor Todd B. Marder grew up in Brooklyn, New York, and received his BSc from the Massachusetts Institute of Technology, Cambridge (USA, 1976), and his PhD from the University of California at Los Angeles (USA, 1981). This was followed by postdoctoral research at the University of Bristol (UK, 1981–1983). Working with the well-renowned inorganic chemists Professor Alan Davison, FRS (BSc), Professor Fred Hawthorne (PhD), and Professor Gordon Stone, CBE, FRS (postdoc) embedded the seeds of curiosity in inorganic chemistry in him, and it initiated his passion for organometallic and boron chemistry, homogeneous catalysis, dynamic NMR studies, and MO calculations. After two years as a visiting research scientist at DuPont Central Research, Wilmington, Delaware (USA), he began his independent academic career at the University of Waterloo (Canada) in 1985, where I first encountered this young, highly enthusiastic, gentleman inorganic chemist. I am proud to be the first PhD student to have graduated from his group.

Within a short span of about 8 years, he was promoted first to Associate Professor and then Full Professor in 1993. Diversity in chemistry was apparent in Todd’s very first collection of young graduate students: indenyl-rhodium chemistry (Ashok Kakkar); Rh and Pt-acetylide chemistry: Pauline Chow, Davit Zargarian (Professor at Université de Montreal, Canada), M.J. Gerald Lesley (Professor at Southern Connecticut State University, USA), Dr. Graham Stringer and Dr. Ian R. Jobe (early postdocs from Oxford and the University of Western Ontario, respectively); metal boryl complexes and metal-catalyzed hydroboration chemistry: Steve Westcott (Professor at Mt. Allison University, Canada). As they say, there is always a woman behind every successful man, and Todd met his wife and a gem of a person, Anne, who was also teaching at the University of Waterloo. These two are a team that continue to support some of the best scientists in their care. In fact, their son, Ian, is an Assistant Professor in Criminology in the Department of Law at Maynooth University, Ireland, so academics run in the family.

With the excellence in chemistry that Todd demanded from himself and his crew, awards started to follow, the first being the Rutherford Memorial Medal for Chemistry (1995), given by the Royal Society of Canada to the leading chemist in Canada under 40 years of age.

After spending 12 years in Canada, Todd was lured by the British way of life, and decided to move from the colonies to the kingdom in 1997, when he accepted the Chair in Inorganic Chemistry at the University of Durham in England. He received the Royal Society of Chemistry Award in Main Group Element Chemistry in 2008 for his contributions to the chemistry of boron and its organometallic compounds and their applications, and a Japan Society for the Promotion of Science (JSPS) Invitation Fellowship (Japan), a Humboldt (Senior) Research Award (Germany), and the Royal Society (UK) Wolfson Research Merit Award, all in 2010. It was finally the lure of German beer, sausages and boron chemistry that brought him to the Institut für Anorganische Chemie, Julius-Maximilians-Universität Würzburg (Germany) in 2012, as Professor and Chair I of Inorganic Chemistry, as well as co-Head of the Institute for Sustainable Chemistry & Catalysis with Boron. Todd also won the Royal Society of Chemistry Organometallic Chemistry Award and was elected to the Bayerische Akademie der Wissenschaften (Bavarian Academy of Sciences) in 2015, Fellow of the American Association for the Advancement of Science (AAAS) in 2018, and Fellow of the European Academy of Sciences (EurASc) in 2019. In 2018, he was also awarded Docteur Honoris Causa, by the Université de Rennes 1, France.

Todd loves varied cultures, food, and his many friends scattered around the world and, prior to the current pandemic, would never miss an opportunity to travel both to contribute and to learn. This has led to several Visiting and similar Professorships in Europe (England, France), Asia (China, Hong Kong, India, Japan), and Australia (David Craig Visiting Professor at the Australian National University, 2014). He has served on the editorial board of several journals including Inorganic Chemistry, Organometallics, the Journal of Organometallic Chemistry, Applied Organometallic Chemistry, Canadian Journal of Chemistry, Crystal Engineering, and Chinese Journal of Chemistry.

Todd has given over 400 invited lectures worldwide, published over 385 papers with an h-index of 88 and around 25,000 citations (WoS). The co-workers he has trained occupy academic (over 35 of them) as well as industrial and other positions all over the world, and their success is his proudest achievement. Indeed, he refers to us as his children, and our academic offspring as his grandchildren, and has even had the pleasure of supervising a postdoc who was also his academic great granddaughter, Marie-Hélène Thibault (Professeure adjointe, Université de Moncton, Canada)!

Todd embraces diversity not only in his group members and numerous local and international collaborators, but also in his research interests [1,2,3,4,5,6,7,8,9,10,11,12,13,14,15,16,17,18,19,20]. For over 30 years, a significant part of his research has focused on metal-catalyzed borylation reactions, metal boryl complexes, mechanisms of borylation and other catalytic reactions [1,2,3,4,5,6], and the development of diboron(4) compounds and their chemistry [7,8,9]. One of the diboron(4) compounds he developed in collaboration with Prof. N. C. Norman (Bristol University), namely B_2_neop_2_, is now available commercially world-wide and is produced in multi-ton quantities. He also works in the fields of conjugated organometallic [10], organoboron [11,12,13,14,15,16] and organic materials [17,18,19] for linear and nonlinear optics, 2-photon excited fluorescent cell imaging [15], DNA/RNA/protein sensing [16], and on crystal engineering using perfluoroarene-arene interactions [20], among other areas, often combining several areas of his research in a single project.

Boron chemistry continues to offer a diverse platform for new directions in catalysts [21,22,23], as well as new cluster [24,25] and other materials [26,27,28,29,30] with varied applications, as indicated in these reviews, some by authors of papers in this issue. Professor Todd B. Marder has contributed significantly to expand the scope of boron chemistry, and it is an honor to celebrate his achievements, and mark his 65th birthday with a Special Issue of *Molecules*: “Boron in Catalysis and Materials Chemistry: A Themed Issue in Honor of Professor Todd B. Marder on the Occasion of His 65th Birthday”. It includes excellent review articles on conjugated N^C-organoboron materials, and tri(boryl)-alkanes and -alkenes as versatile reagents in organic synthesis [31,32], and research articles depicting diversity [33,34,35,36,37,38,39], as noted in the research interests of Professor Marder. We thank all the authors for joining us to congratulate Todd, and for making this issue a successful one through their excellent scientific contributions.
